# Promoting a Syndemic Approach for Cardiometabolic Disease Management During COVID-19: The CAPISCO International Expert Panel

**DOI:** 10.3389/fcvm.2021.787761

**Published:** 2021-12-15

**Authors:** Wael Al Mahmeed, Khalid Al-Rasadi, Yajnavalka Banerjee, Antonio Ceriello, Francesco Cosentino, Massimo Galia, Su-Yen Goh, Peter Kempler, Nader Lessan, Nikolaos Papanas, Ali A. Rizvi, Raul D. Santos, Anca P. Stoian, Peter P. Toth, Manfredi Rizzo, The CArdiometabolic Panel of International experts on Syndemic COvid-19 (CAPISCO)

**Affiliations:** ^1^Cleveland Clinic, Heart and Vascular Institute, Abu Dhabi, United Arab Emirates; ^2^Medical Research Center, Sultan Qaboos University, Muscat, Oman; ^3^Department of Biochemistry, Mohamed Bin Rashid University, Dubai, United Arab Emirates; ^4^IRCCS MultiMedica, Milan, Italy; ^5^Unit of Cardiology, Karolinska Institute and Karolinska University Hospital, University of Stockholm, Stockholm, Sweden; ^6^Department of Biomedicine, Neurosciences and Advanced Diagnostics (Bind), University of Palermo, Palermo, Italy; ^7^Department of Endocrinology, Singapore General Hospital, Singapore, Singapore; ^8^Department of Medicine and Oncology, Semmelweis University, Budapest, Hungary; ^9^Imperial College London Diabetes Centre, The Research Institute, Abu Dhabi, United Arab Emirates; ^10^Second Department of Internal Medicine, Diabetes Center, University Hospital of Alexandroupolis, Democritus University of Thrace, Alexandroupolis, Greece; ^11^Department of Medicine, University of Central Florida College of Medicine, Orlando, FL, United States; ^12^Division of Endocrinology, Diabetes and Metabolism, University of South Carolina School of Medicine, Columbia, IN, United States; ^13^Heart Institute (InCor) University of Sáo Paulo Medical School Hospital, Sáo Paulo, Brazil; ^14^Hospital Israelita Albert Einstein, Sáo Paulo, Brazil; ^15^Faculty of Medicine, Diabetes, Nutrition and Metabolic Diseases, Carol Davila University, Bucharest, Romania; ^16^Cicarrone Center for the Prevention of Cardiovascular Disease, Johns Hopkins University School of Medicine, Baltimore, MD, United States; ^17^Department of Health Promotion, Mother and Child Care, Internal Medicine and Medical Specialties (Promise), University of Palermo, Palermo, Italy

**Keywords:** diabetes, cardiovascular diseases, complications, COVID-19, pandemic, syndemic

## Abstract

Efforts in the fight against COVID-19 are achieving success in many parts of the world, although progress remains slow in other regions. We believe that a syndemic approach needs to be adopted to address this pandemic given the strong apparent interplay between COVID-19, its related complications, and the socio-structural environment. We have assembled an international, multidisciplinary group of researchers and clinical practitioners to promote a novel syndemic approach to COVID-19: the CArdiometabolic Panel of International experts on Syndemic COvid-19 (CAPISCO). This geographically diverse group aims to facilitate collaborative-networking and scientific exchanges between researchers and clinicians facing a multitude of challenges on different continents during the pandemic. In the present article we present our “manifesto”, with the intent to provide evidence-based guidance to the global medical and scientific community for better management of patients both during and after the current pandemic.

## Introduction

There is a bidirectional pathophysiologic relationship between coronavirus disease 2019 (COVID-19) and cardiometabolic diseases, and individuals at risk of the latter require careful consideration as the global pandemic continues to take its toll. Diabetes, obesity, and cardiovascular disease are associated with an increased risk for severe forms of COVID-19 and resulting death (1–6). At the same time, patients with COVID-19 infection are more prone to the development of new-onset diabetes mellitus (7). Investigators from different areas have emphasized the clinical relevance of the increased incidence of diabetes after severe acute respiratory syndrome coronavirus 2 (SARS-CoV-2) infection (8, 9). Furthermore, COVID-19 is associated with cardiovascular injury attributed to heightened inflammation, endothelial dysfunction and microthrombi formation (10–13). Endothelial dysfunction plays an important role in the pathogenesis of COVID-19, particularly in patients with pre-existing hypertension, diabetes, obesity and cardiovascular diseases (14). The endothelium, and particularly pulmonary endothelium, seems to be a key target organ in COVID-19 patients and its dysfunction has been shown to cause an impaired organ perfusion that can generate acute myocardial injury, renal failure, and a procoagulant state resulting in thromboembolic events (14–16).

It has been shown that SARS-CoV-2 can induce several pro-inflammatory cytokines (17) and that patients with severe COVID-19 develop a “cytokine storm syndrome” (18). Since these first observations it is become clear that the same cytokines that induce aberrant endothelial function may also trigger the acute phase response, which, in combination with local endothelial dysfunction, can lead to clinical consequences (14); indeed, inflammatory cytokines have a major role in both diabetes and cardiovascular diseases (19). Other authors have shown that COVID-19 is associated with myocardial damage such as myocarditis, arrhythmia and reduced left ventricular ejection fraction (20), all of which are associated to increased mortality risk (21). Plasma cardiac biomarkers, such as high sensitivity troponin, creatine kinase and N-terminal pro-B-type natriuretic peptide, are also associated with COVID-19 severity in adults and children (22, 23).

Beyond the direct effect of COVID-19 on the cardiovascular system and metabolic homeostasis, subjects with pre-existing cardiometabolic issues appear to be at a significantly higher risk of complications owing to reduced physical activity, altered eating behaviors, and lack of access to healthcare (24). This encapsulates the so-called “indirect” impact of COVID-19 and indeed a higher incidence of cardiovascular complications and fatalities has been documented secondary to the pandemic, for example in Italy (25). Even in New York City, emergency calls for cardiac arrests rose exponentially in the weeks when COVID-19 infections approached their zenith (26). Beyond deaths attributed directly to COVID-19, a large contribution to the excess mortality reported (27) is attributable to the indirect factors, including the disruption of the proper management of many clinical conditions–including cardiometabolic diseases–by the rapid conversion of entire hospitals or clinical units to deliver COVID-19-specific care (28).

This situation has been exacerbated in some geographical areas by increased unemployment, economic collapse, and widespread poverty (29). Indeed, increasing socio-economic disparities have come to the forefront in many populations during the pandemic, rendering people more vulnerable to economic, nutritional, social, and medical insecurity, particularly during prolonged periods of necessary government-imposed restrictions or lockdowns (30). In addition, the spread of the virus and the related complications and fatalities have been facilitated among subjects with the poorest socio-economic conditions and those living in overcrowded areas (29–31). Socio-economic inequalities are of increasing relevance during the ongoing vaccination campaigns as they contribute to disparities in care across different ethnic populations and geographical areas (32).

## Importance of a Syndemic Approach

The enormous efforts by many different organizations and individuals involved in the fight against COVID-19 have had some success in many parts of the world. However, this has not been universal and progress has been slow in many other regions. We believe that a syndemic approach needs to be adopted for addressing this pandemic (33) given the strong apparent interplay between COVID-19, its related complications, and the socio-structural environment. The term *syndemic* (from ancient Greek: *syn*, together; *demos*, people) emphasizes the relevance of biological, social, economic, and environmental factors in the health of individuals and populations (29). Physicians have an obligation to understand their patients' social, economic, and environmental situations and to utilize the tools available in existing health systems to improve their access to care. It is also expected that many health systems will continue to be under significant economic pressure, which may contribute to a reduced quality of care for patients with chronic conditions.

Another challenge is represented by the so-called *long-COVID syndrome*, which is a clinical condition present in subjects who have either recovered from COVID-19 but still report lasting effects of the infection or have had the usual symptoms for far longer than would be expected (34). Of increasing current interest are the neurological and neuropsychiatric complications (35), since several studies have reported a broad spectrum of symptoms, from the milder manifestations of memory loss, sleep disorders and impaired concentration to more serious cognitive decline, major depression or persistent delirium (36). Thus, there is an urgent need to better understand the long-term effects of COVID-19 on brain function, behavior and cognition. As a component of a holistic approach to the management of patients with COVID-19, mental health assessment should be included.

## Capisco: An International, Multidisciplinary Collaboration

**Figure 1:**
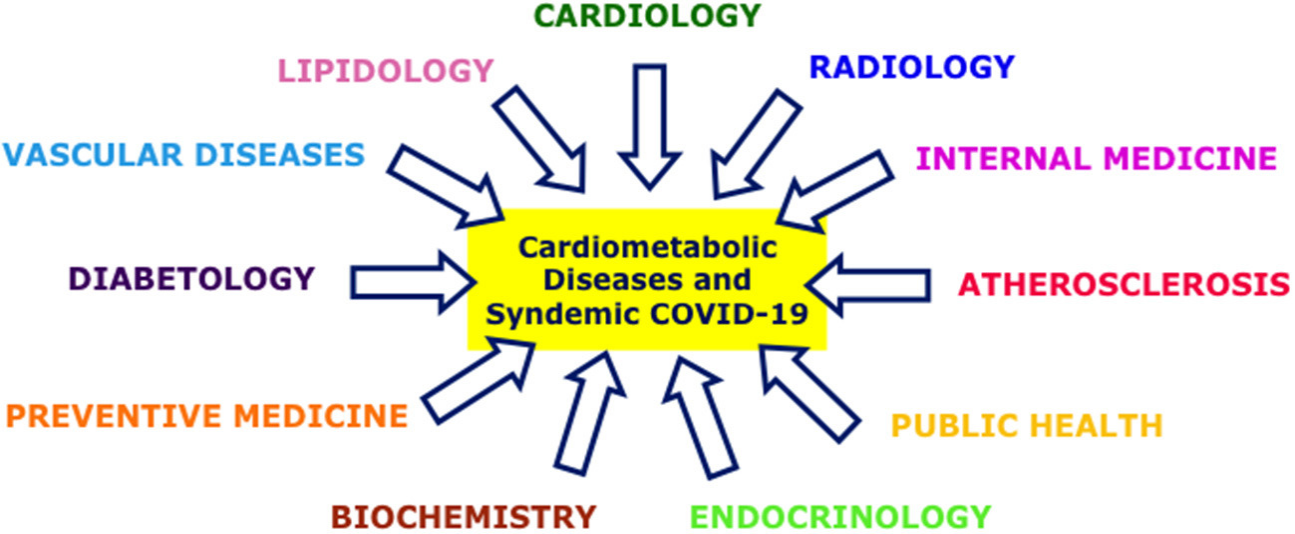
A novel multi-disciplinary syndemic approach to cardiometabolic diseases during COVID-19.

We believe it is crucial to improve interactions between specialists working in different disciplines, since insufficient cooperation has contributed to the indirect impact of COVID-19 (37). Furthermore, the pandemic has adversely medical education in many ways, for example owing to shifts to distance-learning modalities and decline in clinical clerkship due to the cancelation of routine patient appointments and surgical procedures and a transition to greater use of telemedicine (38). In response to these challenges we have recently assembled the Cardiometabolic Panel of International Experts on Syndemic COVID-19 (CAPISCO), a group of international researchers and clinical practitioners from many different disciplines including (but not limited to) diabetology, endocrinology, cardiology, lipidology, internal medicine, radiology, preventive medicine, public health and biochemistry–providing a multi-disciplinary representation in a novel approach to COVID-19 (Figure 1). We also emphasized geographical diversity when convening the group in order to facilitate collaborative-networking and scientific exchanges between researchers and clinicians facing a multitude of challenges in different continents during the pandemic.

Members of CAPISCO intend to collaboratively investigate:

1. how patients with cardiometabolic diseases and its complications are currently being managed and treated and the extent to which the pandemic impacts proper management, with a view to identifying, categorizing and defining innovative strategies to overcome potential barriers and disparities;
2. why differences in COVID-19 mortality rates have been reported among various countries, beyond the prevalence of the disease *per se*, with the aim to elucidate the social, economic and environmental factors potentially impacting clinical outcomes;
3. whether telemedicine is a reliable and useful tool to deliver high-quality patient care in light of experience gained during the pandemic; across different geographical areas we aim to investigate: what was successfully implemented and how, what was not successfully implemented and why, and what still needs to be improved;
4. how to assess the burden and late consequences of delayed management of cardiometabolic disease and other conditions due to COVID-19.

Ultimately, the overarching aim of CAPISCO is to give evidence-based guidance for the management of patients with cardiometabolic diseases during and after COVID-19 based on a syndemic approach. In terms of methodology, we plan to use a systematic approach, including systematic literature searches, formal quality-grading and analysis of collected studies, resulting in graded levels of recommendations. We also intend to make a roadmap plan of further research avenues once the data from the above indicated tools becomes available; this may also involve cooperation with health authorities and other international organizations.

## Conclusions

In conclusion, we believe it is crucial to view COVID-19 through a syndemic lens to properly tackle the interlinked public health, medical, social and economic challenges that amplify each other in this crisis. The acronym of our expert panel, CAPISCO, is meaningful, since the word “*capisco*” means in Italian, “*I understand*”. CAPISCO contributions will promote a holistic approach for all patients with cardiometabolic diseases based on solid, validated scientific research and clinical expertise. We hope that physicians around the world will be able to use them to help benefit clinical care, follow-up, and monitoring of their patients during and after the COVID-19 pandemic.

## Data Availability Statement

The original contributions presented in the study are included in the article/supplementary material, further inquiries can be directed to the corresponding author.

## Author Contributions

This article is the result of three expert panel meetings conducted virtually between May and July 2021. MR and WA, co-chairs of CAPISCO, prepared the first draft of this article, which was first critically discussed and reviewed with AR, AC, and AS, and then extensively reviewed by all the other members. All authors have equally contributed to the final manuscript and are listed alphabetically.

## Conflict of Interest

The authors declare that the research was conducted in the absence of any commercial or financial relationships that could be construed as a potential conflict of interest.

## Publisher's Note

All claims expressed in this article are solely those of the authors and do not necessarily represent those of their affiliated organizations, or those of the publisher, the editors and the reviewers. Any product that may be evaluated in this article, or claim that may be made by its manufacturer, is not guaranteed or endorsed by the publisher.
